# Assessment of transposition of the great arteries associated with multiple malformations using dual-source computed tomography

**DOI:** 10.1371/journal.pone.0187578

**Published:** 2017-11-20

**Authors:** Lin-jun Xie, Li Jiang, Zhi-gang Yang, Ke Shi, Hua-yan Xu, Rui Li, Kai-yue Diao, Ying-kun Guo

**Affiliations:** 1 Department of Radiology, West China Hospital, Sichuan University, Chengdu, Sichuan, China; 2 Department of Radiology, Key Laboratory of Obstetric & Gynecologic and Pediatric Diseases and Birth Defects of Ministry of Education, West China Second University Hospital, Sichuan University, Chengdu, Sichuan, China; New York Medical College, UNITED STATES

## Abstract

**Purpose:**

To determine the value of dual-source computed tomography (DSCT) in depicting the morphological characteristics and diagnosing the associated malformations for patients with transposition of the great arteries (TGA) before surgery.

**Materials and methods:**

Twenty-five patients with TGA who underwent DSCT and transthoracic echocardiography (TTE) examination were retrospectively reviewed. The morphological types of TGA, the spatial relationship between the pulmonary artery and the aorta, as well as coronary artery-associated abnormalities were assessed by DSCT. In contrast to TTE, the diagnostic accuracy of associated malformations on DSCT were analyzed and calculated with reference to surgical or digital subtraction angiography (DSA) findings. Effective doses (EDs) were also calculated.

**Results:**

Among the 25 patients, 12 (48%) had ventricular septal defects and left ventricular outflow tract stenosis. Sixteen patients (16/25, 64%) had great arteries with an oblique spatial relationship on DSCT. In addition, we found seven patients (7/25, 28%) with coronary artery malformation, including five with an abnormal coronary origin and two with signs of a myocardial bridge. According to DSA or surgical findings, DSCT was superior to TTE in demonstrating extracardiac anomalies (sensitivity, anomalies of great vessels: 100% vs. 93.33%, other anomalies: 100% vs. 46.15%). The mean estimated ED for those aged <10 years was <2 mSv (1.59 ± 0.95 mSv).

**Conclusions:**

DSCT can achieve an overall assessment of patients with TGA, including any associated malformations as well as the identification of the spatial relationship of the great arteries. DSCT can therefore be considered as an alternative imaging modality for surgical decision making.

## Introduction

Transposition of the great arteries (TGA) is a rare anomaly with an estimated prevalence of 5%–7% among all congenital heart diseases (CHD) [1]. The main clinical manifestations of TGA are cyanosis and dyspnea [2]. TGA has a normal atrioventricular connection but a ventriculoarterial discordance, in which the pulmonary artery arises from the morphological left ventricle and the aorta arises from the morphological right ventricle (RV) [3]. TGA is considered to account for a variety of organ damage in infants because of the decreased level of brain oxygen [4,5]. Thus, a delayed diagnosis and intervention will expose patients to increased risk [6]. It is vital that the choice of operation is based on the knowledge of the spatial relationship of the great arteries and the coronary artery abnormalities. Accurate and comprehensive evaluation of TGA and associated abnormalities is thus critical before surgery.

With high temporal and spatial resolutions, DSCT can comprehensively evaluate anatomical characteristics. DSCT can be currently utilized to obtain images with good quality at a lower radiation dose, without considering the effects of heart rate for pediatric patients [7,8]. DSCT is now recognized as a routine examination method for various kinds of CHD, especially complex CHD [9,10,11]. However, research examining DSCT use in diagnosing associated malformations in TGAs is currently limited [12]. We therefore chose to evaluate the value of DSCT in depicting the morphological characteristics and diagnosing the associated malformations of TGA patients for making decisions on surgery types.

## Materials and methods

### Study population

A total of 27 patients were enrolled in our hospital between July 2010 and December 2016 with a diagnosis of TGA according to the diagnostic criteria of the ACC/AHA 2008 guideline [13]. All patients underwent TTE and DSCT examinations. The exclusion criteria were incomplete clinical history and poor image quality (n = 2). Finally, a total of 25 patients remained, including 15 males and 10 females (mean age: 7.72 ± 11.07 years, ranging from 3 months to 37 years). Fourteen patients underwent corrective surgery and four patients underwent palliative surgery. The institutional review board of our hospital approved this study (No. 14–163). All patients (or guardians) signed informed consent for CT examinations, including the use of iodine contrast and radiation exposure. All patient-sensitive information was treated with full confidentiality and used solely for the purposes of this study.

### Dual-source CT protocol

We used a dual-source CT system (Somatom Definition; Siemens Medical Solutions, Forchheim, Germany). Adults and children (>6 years of age) were required to hold their breath while scanning. During the scan, the patients aged <6 years of age were sedated using chloral hydrate (concentration: 10%, 0.5 ml/kg). A retrospective ECG-gated cardiac CT scan was used in this study with the following acquisition parameters: tube current, automatic adjustment; tube voltage, 80–120 kV; gantry rotation time, 0.28 s; and pitch, 0.2–0.5. The ECG-pulsing window was set to “auto.” The nonionic contrast material (iopamidol; 370 mg/ml, Bracco, Sine Pharmaceutical Corp., Ltd., Shanghai, China) was injected via peripheral veins in the elbow or back of the hand at a rate of 1.5–2.5 ml/s, followed by 20 ml of saline solution. The injected volume was calculated according to the body weight (1.5 ml/kg). The acquisition range was from the thoracic inlet to 2 cm below the diaphragm level. A region of interest (ROI) was placed on the descending aorta. The ROI threshold was set to 100 HU. After contrast medium injection, the scan was automatically triggered after a delay of 5 s when the ROI attenuation threshold reached 100 HU.

### Image analysis

The image data were analyzed using a workstation (Syngo; Siemens Medical System, Forchheim, Germany). Image reconstruction was performed using a 0.75-mm slice thickness, including multiplanar reformatting (MPR), and volume rendering (VR). The cardiovascular structures and extracardiac structures were evaluated on reconstructed images by two experienced radiologists. If the results were reported differently, a consensus was established through negotiation by the two radiologists. Both radiologists were blinded to the patient's TTE findings.

We used an imaging analysis method according to the Van Praagh segmental classification system [14]. First, the morphological right and left atria and ventricles must be identified. That requires evaluation of the atrioventricular connection. At the same time, the intracardiac anomalies were also assessed. According to both a ventricular septal defect and left ventricular outflow tract stenosis [15], TGA is divided into four morphological categories: TGA with an intact ventricular septum (TGA-IVS), TGA with an intact ventricular septum and left ventricular outflow tract stenosis (TGA-IVS-LVOTO), TGA with ventricular septal defect (TGA-VSD), and TGA with a ventricular septal defect and left ventricular outflow tract stenosis (TGA-VSD-LVOTO). Next, the visceroatrial situs and ventricular loop orientation were determined from the positions of the cardiac chambers. With this information, the spatial relationship of the great arteries as well as the coronary artery anomalies can be established. The spatial relationship of the pulmonary artery and the aorta was observed on an axial image at the level of the aortic valve, including the oblique, anteroposterior, and side-by-side patterns. To determine the origin of coronary arteries, we used the classic Leiden typing method. It places the observer in the noncoronary sinus, looking toward the pulmonary trunk [16]. From this position one sinus is always to the right of the observer (sinus 1) and the other sinus is always to the left of the observer (sinus 2). This definition holds regardless of the relationship of the great arteries toward each other. The coronary origin is described as the sinus and coronary artery. For example, if the left anterior descending (LAD) and left circumflex (LCx) arise from sinus 1 and the right coronary artery (RCA) arises from sinus 2, the coronary origin is termed as 1L,Cx;2R.

With reference to the surgical results, TGA with malformations can be divided into three categories, including anomalies of the great vessels, intracardiac anomalies, and other cardiac anomalies. Anomalies of the great vessels included pulmonary artery anomalies, pulmonary vein anomalies, a right aortic arch, and a persistent left superior vena cava. Intracardiac anomalies included left ventricular outflow tract stenosis, atrial septal defect, ventricular septal defect, biscuspid pulmonary valve, and patent ductus arteriosus. Other anomalies were referred to as aortopulmonary collateral vessels. With the surgical results as the reference standard, we compared the effectiveness of DSCT and TTE for identifying the combined malformations.

### Transthoracic echocardiography

All patients underwent transthoracic echocardiography with use of a Philips SONOS 7500 ultrasound system (Philips Medical Systems, Bothell, WA). Two-dimensional, M-mode, and Doppler echocardiography were performed according to the guidelines of the American Society of Echocardiography [17]. An experienced echocardiographic technician completed the examination and was blinded to the results of the other study.

### Radiation dose estimation

The machine automatically provides reports on the volume CT dose index (CTDIvol) and dose-length product. The effective doses (EDs) are calculated using the conversion coefficients provided by the 2007 recommendations of the International Commission on Radiological Protection. This states the following coefficients according to age: 0.039, 0.026, 0.018, 0.012, and 0.014 for children aged <4 months, 4–12 months, 1–6 years, 6–10 years, and those aged >10 years of age, respectively [18].

### Statistical analysis

The data were analyzed using SPSS software for Mac (version 24.0, SPSSInc., Chicago, IL, USA). Sensitivity, specificity, positive predictive value, and negative predictive value were calculated for intracardiac anomalies, anomalies of great vessels, and other cardiac anomalies comparing DSCT and TEE. Categorical variables were presented as numbers and percentages. Continuous variables were expressed as means ± standards deviations.

## Results

### Baseline characteristics

**Fig 1:**
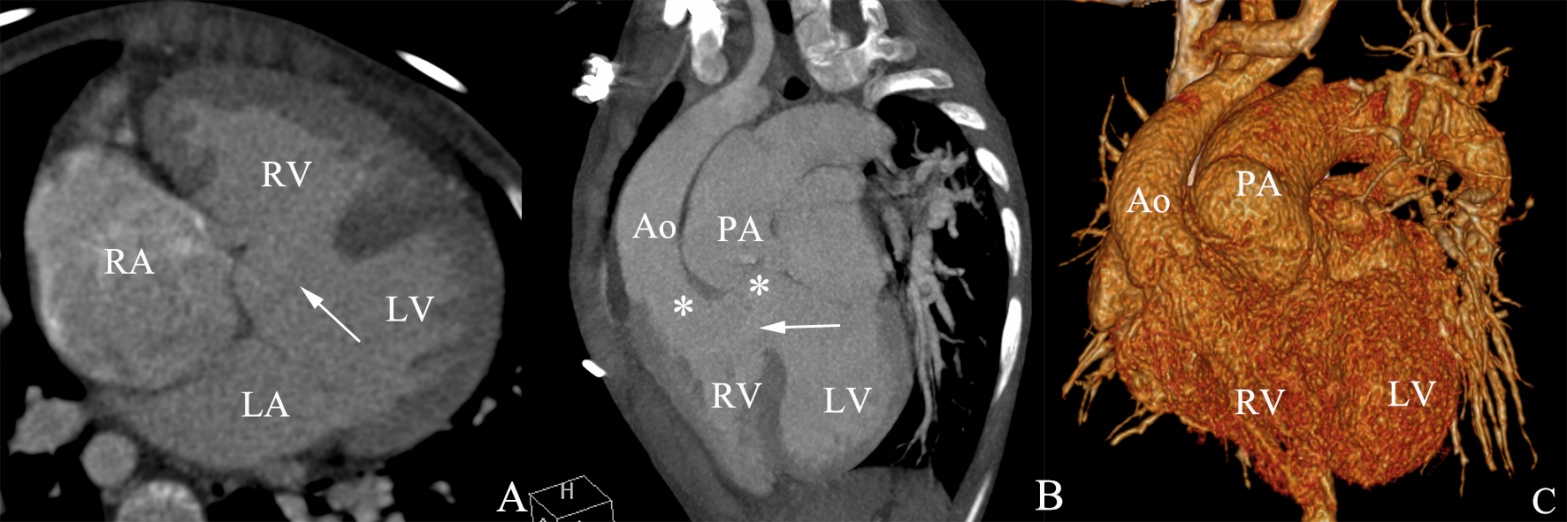
TGA with VSD in a male aged 11 years. (A). The axial image shows a large ventricular septal defect. (B) The multi planar reformatted image shows the pulmonary artery arises from the morphological left ventricle and the aorta arises from the morphological right ventricle. (C) The volume-rendered image shows the spatial relationship of the aorta and the pulmonary artery. LV, left ventricle; RV, right ventricle; Ao, aorta; PA, pulmonary artery; VSD, ventricular septal defect.

**Fig 2:**
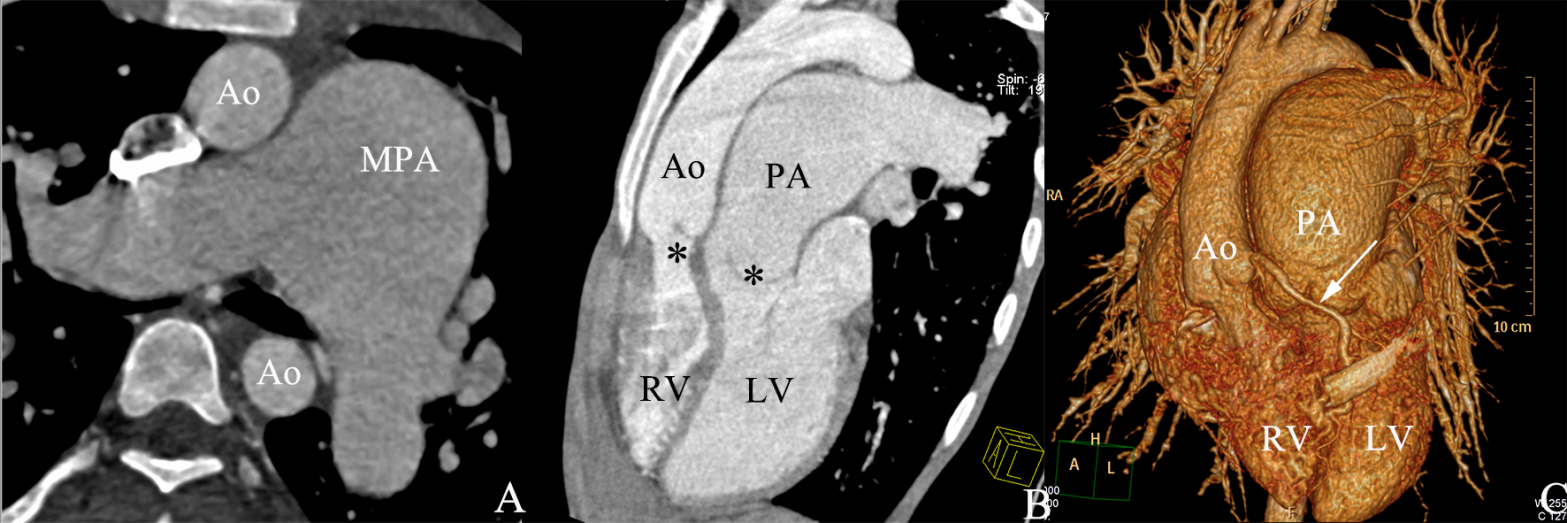
TGA with main pulmonary artery aneurysms in a male aged 25 years. (A) The axial image shows main pulmonary artery aneurysms expansion. (B) The multi planar reformatted image shows the pulmonary artery arises from the morphological left ventricle and the aorta arises from the morphological right ventricle. (C) The volume-rendered image shows the spatial relationship of the aorta and pulmonary artery. LV, left ventricle; RV, right ventricle; Ao, aorta; PA, pulmonary artery; MPA, main pulmonary artery.

Of all the 25 patients, 22 (88%) pediatric patients had cyanotic symptoms and dyspnea. A New York Heart Association class ≥III was present in 11 patients (44%). The incidence according to morphological type was as follows: 12 cases (48%) of TGA-VSD-LVOTO (Fig 1), eight (32%) of TGA-VSD, four (16%) of TGA-IVS (Fig 2), and one (4%) of TGA-IVS-LVOTO (Table 1).

**Table 1:** Patient characteristics (n = 25).

Characteristic	
Gender (male)	15 (60%)
Mean age at DSCT (yrs)	7.72±11.07
Body surface area (m^2^)	0.68±0.38
New York Heart Association class	
I/II	14
III/IV	11
Heart rate (bpm)	113±25
Systolic blood pressure at rest (mm Hg)	96±16
Diastolic blood pressure at rest (mm Hg)	59±12
Types of TGA	
TGA-IVS	4 (16%)
TGA-IVS-LVOTO	1 (4%)
TGA-VSD	8 (32%)
TGA-VSD-LVOTO	8 (32%)

Note: TGA-IVS: TGA with intact ventricular septal; TGA-IVS-LVOTO: TGA with intact ventricular septal and left ventricular outflow tract stenosis; TGA-VSD: TGA with ventricular septal defect; TGA-VSD-LVOTO: TGA with ventricular septal defect and left ventricular outflow tract stenosis.

### The spatial relationship of the great arteries and the coronary artery anomalies

**Fig 3:**
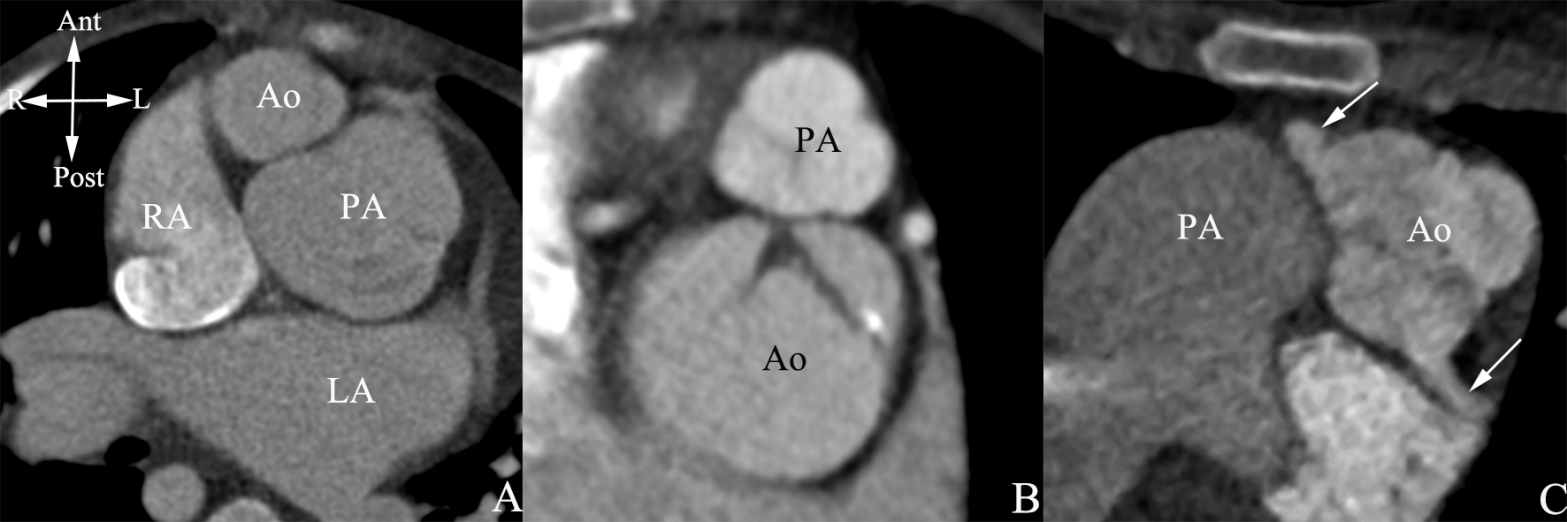
The spatial relationship of the aorta and the pulmonary artery. (A) Oblique position. (B) Anterior-posterior position. (C) Side-by-side position. White arrow referred to the origin of coronary artery. Ant, anterior; Post, posterior; R, right; L, left.

**Fig 4:**
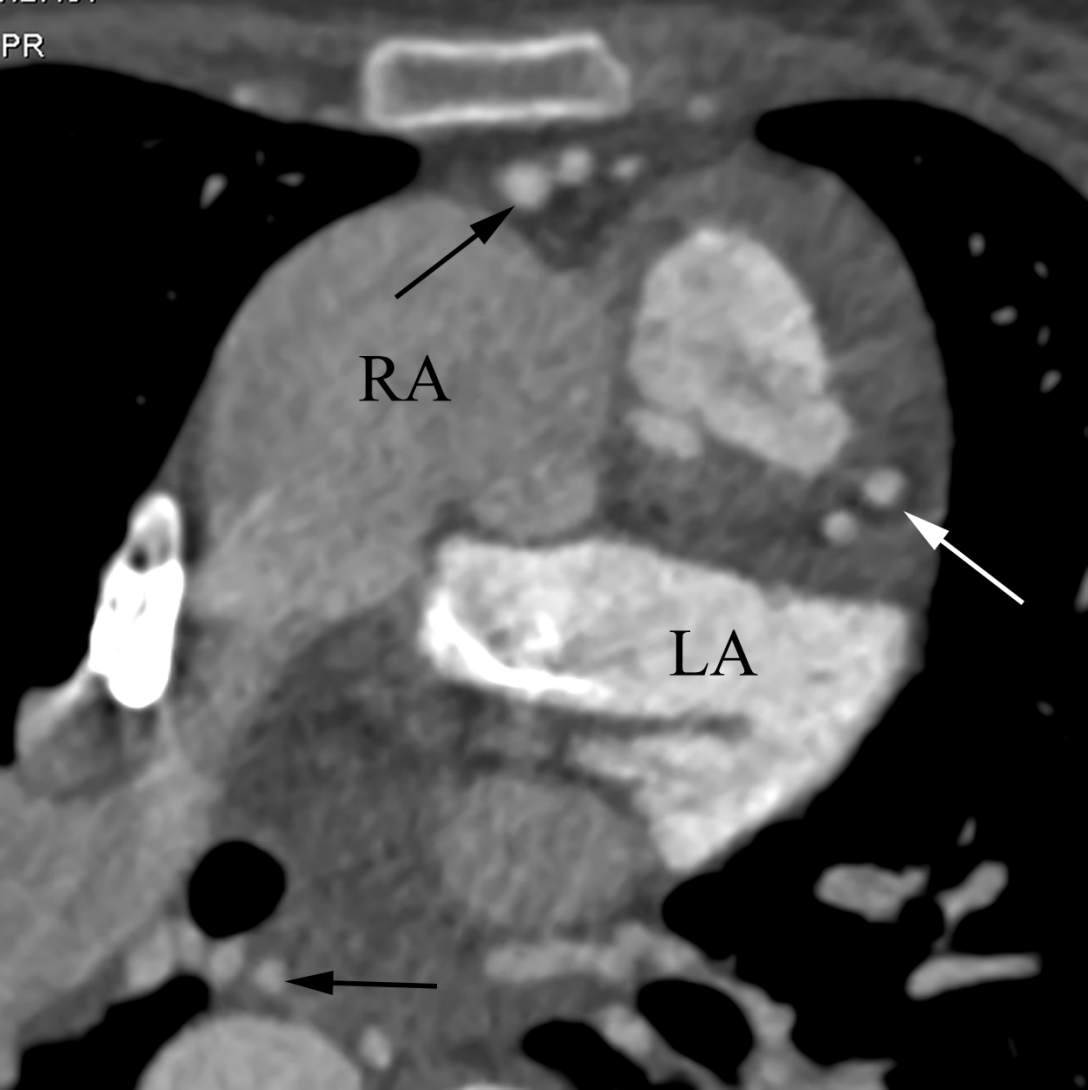
The myocardial bridge. The white arrow shows a myocardial bridge. The black arrow shows a lot of aortopulmonary collateral vessels.

Sixteen patients (16/25, 64%) showed an oblique spatial relationship of the great arteries according to DSCT, whereas seven patients (7/25,28%) showed an anteroposterior arrangement, with the remaining two (2/25, 8%) showing a side-by-side (Fig 3) arrangement. A total of 20 patients (20/25, 80%) presented with the origin of the coronary artery in the 1L,Cx;2R pattern. In this type, 11 patients presented with an oblique arrangement of the great arteries, seven patients had an anteroposterior arrangement, while one had a side-by-side arrangement (Table 2). The remaining patterns of the origin of the coronary artery include 1LR;2Cx (1 case), 1L;2RCx(1 case), 2RLCx(1 case) and 1R;2LCx(2 cases). In addition to the origin of coronary artery, we found two cases having a myocardial bridge (Fig 4).

**Table 2:** The spatial relationship of the great arteries and the coronary artery origin of TGA patients.

Coronary artery anatomy	The spatial relationship of the great arteries		
	Oblique	Anterior-posterior	Side-by-side
Usual (1L,Cx;2R)	12	7	1
Other			
1L,R; 2Cx	1	0	0
1L; 2R,Cx	1	0	1
2R,L,Cx	1	0	0
1R; 2L,Cx	1	0	0

Note: Cx, circumflex artery; L, left anterior descending; R, right coronary artery

### Associated multiple malformations

On the basis of the surgical or DSA findings, there were a total of 80 associated malformations in the 19 TGA patients (Table 3). The sensitivity and specificity for diagnosing TGA associated with intracardiac anomalies were 83.02% and 100% for DSCT and 94.34% and 97.62% for TTE, respectively (Table 4). DSCT missed atrial septal defects in three cases (3/14), bicuspid pulmonary valves in three cases (3/5), and patent ductus arteriosus in three cases (3/6). The sensitivity and specificity for diagnosing TGA associated with anomalies of the great vessels were 100% and 100% for DSCT and 93.33% and 97.62% for TTE, respectively. The sensitivity and specificity for diagnosing TGA associated with other anomalies were 100% and 100% for DSCT but were 46.15% and 100% for TTE, respectively. TTE missed the detection of pulmonary artery anomaly in one case (1/6), pulmonary vein anomaly in one case (1/1), aortopulmonary collateral vessels in seven cases (7/12), and patent ductus arteriosus in one case (1/13); in addition, one case with patent ductus arteriosus was misdiagnosed.

**Table 3:** A summary of the findings obtained with DSCT and TTE (n = 19).

Associated malformations	Surgical results	DSCT findings				TTE findings			
		TP	FN	TN	FP	TP	FN	TN	FP
The anatomy of left ventricular outflow tract stenosis	14(56%)	14	0	5	0	14	0	5	0
Ventricular septal defect	14(56%)	14	0	5	0	14	0	5	0
Atrial septal defect	14(56%)	11	3	5	0	14	0	5	0
Patent ductus arteriosus	6(24%)	3	3	13	0	5	1	12	1
Bicuspid pulmonary valve	5(20%)	2	3	14	0	3	2	14	0
Persistent left superior vena cava	4(16%)	4	0	15	0	4	0	15	0
Pulmonary artery anomalies	6(24%)	6	0	13	0	5	1	13	0
Right aortic arch	5(20%)	5	0	14	0	5	0	13	1
Aortopulmonarycollateral vessels	12(48%)	12	0	7	0	5	7	7	0

Note: TP, true positive finding; FN, false negative finding; TN, true negative finding; FP, false positive finding

**Table 4:** The diagnostic accuracies of DSCT and TTE according to anomalies categories.

Anomalies categories	DSCT				TTE			
	Sen	Spec	PPV	NPV	Sen	Spec	PPV	NPV
Intra-cardiac anomalies	83.02%	100%	100%	82.35%	94.34%	97.62%	98.04%	93.18%
Anomalies of great vessels	100%	100%	100%	100%	93.33%	97.62%	93.33%	97.62%
Others anomalies	100%	100%	100%	100%	46.15%	100%	100%	78.13%

Note: Sen, sensitivity; Spec, specificity; PPV, positive predictive value; NPV, negative predictive value; Others anomalies: aortopulmonary collateral vessels

### Radiation dose estimation

The mean ED for patients aged 0–4 months was 1.31 ± 1.07 mSv, for those aged 4–12 months was 1.58 ± 0.81 mSv, for those aged 1–6 years was 1.31± 0.39 mSv, for those aged 6–10 years was 2.17 ± 1.54 mSv, and for those aged ≥10 years was 6.05 ± 3.13 mSv. The estimated mean ED for those aged <10 years was 1.59 ± 0.95 mSv.

## Discussion

TGA is one of the most common causes of cyanotic CHD, the prevalence is 0.2–0.3 per 1000 live births, often accompanied with multiple malformations and coronary artery anomalies [19]. Because there is inadequate oxygen transportation between the pulmonary and systemic circulations in the body, these infants show cyanosis and dyspnea. Early diagnosis as well as the appropriate surgical therapy is particularly important for a child’s growth.

Traditionally, DSA has been regarded as the gold standard imaging modality for TGA before surgery. However, the high radiation dose and the invasive operation are the major limitations of DSA. At present, TTE is the first-line clinical choice, because of its portable size and moderate price although this noninvasive technology is limited by the small acoustic window and the operator's experience. Cardiac magnetic resonance imaging can evaluate both the anatomy and the function of those with congenital heart disease [20]; however, because of the long examination time and higher cost, CMR is challenging in the clinical setting, especially in uncooperative children. With a high temporal resolution of 83 ms, DSCT significantly decreases the impact of a high heart rate on cardiac data acquisition. Advances in DSCT have increased the feasibility of diagnosing complex CHD; hence, we now use DSCT preoperatively to evaluate the associated anomalies in patients with TGA.

In our study, DSCT had a high sensitivity in detecting anomalies of the great vessels (100% vs. 93.33%) and detection of other anomalies (100% vs. 46.15%) compared with traditional TTE. The reason for the lower sensitivity levels with TTE might include the acoustic window and the experience of the operator, especially when diagnosing the great vessels and separate thoracic anomalies.

In this study, TTE appears to be superior to DSCT for detecting intracardiac anomalies. DSCT missed several tiny anomalies such as atrial septal defect (3/14), patent ductus arteriosus (3/6), and bicuspid pulmonary valve (3/5). With regard to this aspect, DSCT diagnosis is based on static, two-dimensional images, which makes it difficult to accurately assess many subtle intracardiac structures, especially for patients whose diagnosis depends on hemodynamic analysis. On the contrary, Doppler ultrasound blood flow detection technology is particularly suited to diagnosing subtle diseases of the circulatory system [21].

Traditionally, TGA is also known as D-TGA. The “D” signifies that the aorta is located in an anterior and rightward position relative to the pulmonary artery, which is also known as the oblique relationship [16]. However, the term of D-TGA is controversial. Despite the oblique relationship being the most common type; previous studies also found other types including the anteroposterior and side-by-side patterns. According to our research, the oblique relationship is the most common type, accounting for 64% of cases, with the anteroposterior pattern accounting for 28% and the remaining 8% the side-by-side pattern. DSCT has the ability to offer an intuitive and detailed spatial examination of the great arteries before surgery.

Because the great arteries and ventricles join incorrectly, the origin and course of the coronary artery is different from that in normal people. During the arterial switch operation for TGA, reimplantation of the coronary arteries presents the principal difficulty in the surgical procedure, particularly in cases where there are abnormalities of the coronary origin or its course. It is vital to accurately assess the coronary artery before surgery. The most “usual” pattern of coronary artery origin presents with the LAD and left circumflex arising from sinus 1, with the RCA arising from sinus 2 [22]. Similar to previous reports, 80% (20/25) patients presented with this pattern in our study. We also found 20% (5/25) patients with the rarer patterns of coronary origin. Because of the higher spatial resolution than TTE and the powerful post-processing techniques such as MPR and VR, DSCT is beneficial for assessing the origin and orientation of abnormal coronary arteries in children with TGA [23].

Previous research demonstrated that radiation exposure can cause some side effects [24]. The pediatric population is sensitive to ionizing radiation, and radiation exposure will increase their potential cancer risk [24]. According to the “ALARA” (as low as reasonable achievable) principle, we adopt the most commonly used method to minimize the radiation dose, including a constant tube voltage and tube current reduction scanning methods. Respiratory training for larger children is applicable, thereby avoiding unnecessary repeated exposure. Phalla et al. [25] found that the median ED of a 64-slice CT in children with CHD was 4.5± 0.5 mSv. The radiation dose was greatly decreased in our series (approximately 2 mSv, 1.59 ± 0.95 mSv) compared with a 64-slice CT. Furthermore, the radiation dose can be sharply decreased in further studies by using prospective ECG-gated protocol, personalized scanning solutions, among other techniques.

We acknowledge the following limitations in our study. First, TGA is a rare congenital heart disease, with a prevalence of 0.2–0.3 per 1000 live births [22]. Our single-center study collected 25 patients during the past 7 years. The sample size was relatively small, so larger samples are necessary for confirming the clinical significance of preoperative TGA evaluation with DSCT. Second, we did not include patients with the corrected TGA. We did not discuss these two diseases in this one study because they have different morphological and hemodynamic characteristics. Third, we only discussed the pros and cons of preoperative TGA assessment with DSCT and TTE, but the clinical significance need be further observed with long-term follow-up.

In conclusion, DSCT can precisely depict the spatial relationship between the great arteries and diagnose the associated malformations for patients with TGA. The combination of DSCT and TTE can provide a safe and effective imaging alternative modality for preoperative evaluation.

## Supporting information

S1 Table The spatial relationship of the great arteries and the coronary artery anomalies. Note: Cx, circumflex artery; L, left anterior descending; R, right coronary artery.
(DOCX)
