# Supplementary material for: Assessment of transposition of the great arteries associated with multiple malformations using dual-source computed tomography
Source: PLoS One. 2017 Nov 20;12(11):e0187578. doi: 10.1371/journal.pone.0187578 (PMC5695805; doi:10.1371/journal.pone.0187578)
Supplement: S1 Table — Note: Cx, circumflex artery; L, left anterior descending; R, right coronary artery. (DOCX) [file pone.0187578.s001.docx]

**S1 Table. The spatial relationship of the great arteries and the coronary artery anomalies**

| Number | Oblique | Anterior-posterior | Side-by-side | Coronary artery anatomy |  |
| --- | --- | --- | --- | --- | --- |
| 1 |  |  | √ | 1L,Cx;2R | myocardial bridge |
| 2 | √ |  |  | 1L,Cx;2R |  |
| 3 | √ |  |  | 1L,Cx;2R |  |
| 4 |  | √ |  | 1L,Cx;2R |  |
| 5 |  | √ |  | 1L,Cx;2R |  |
| 6 | √ |  |  | 1L,R;2Cx |  |
| 7 | √ |  |  | 1L,Cx;2R |  |
| 8 | √ |  |  | 2R,L,Cx |  |
| 9 |  | √ |  | 1L,Cx;2R |  |
| 10 | √ |  |  | 1L,Cx;2R | myocardial bridge |
| 11 | √ |  |  | 1L,Cx;2R |  |
| 12 | √ |  |  | 1L,Cx;2R |  |
| 13 |  |  | √ | 1L;2R,Cx |  |
| 14 |  | √ |  | 1L,Cx;2R |  |
| 15 | √ |  |  | 1L,Cx;2R |  |
| 16 |  | √ |  | 1L,Cx;2R |  |
| 17 | √ |  |  | 1L,Cx;2R |  |
| 18 | √ |  |  | 1L,Cx;2R |  |
| 19 | √ |  |  | 1L,Cx;2R |  |
| 20 | √ |  |  | 1L,Cx;2R |  |
| 21 |  | √ |  | 1L,Cx;2R |  |
| 22 |  | √ |  | 1L,Cx;2R |  |
| 23 | √ |  |  | 1R;2L,Cx |  |
| 24 | √ |  |  | 1L,Cx;2R |  |
| 25 | √ |  |  | 1L;2R,Cx |  |

Note: Cx, circumflex artery; L, left anterior descending; R, right coronary artery
